# Gender dimension in cardio-pulmonary continuum

**DOI:** 10.3389/fcvm.2022.916194

**Published:** 2022-08-08

**Authors:** Leah Hernandez, Agne Laucyte-Cibulskiene, Liam J. Ward, Alexandra Kautzky-Willer, Maria-Trinidad Herrero, Colleen M. Norris, Valeria Raparelli, Louise Pilote, Peter Stenvinkel, Karolina Kublickiene, the GOING-FWD Consortium

**Affiliations:** ^1^Division of Renal Medicine, Department of Clinical Science, Intervention and Technology, Karolinska Institutet, Stockholm, Sweden; ^2^Department of Nephrology, Lund University, Skåne University Hospital, Malmö, Sweden; ^3^Department of Forensic Genetics and Forensic Toxicology, National Board of Forensic Medicine, Linköping, Sweden; ^4^Division of Endocrinology and Metabolism, Department of Medicine III, Medical University of Vienna, Vienna, Austria; ^5^Clinical and Experimental Neuroscience, Institutes for Aging Research and Bio-Health Research of Murcia, School of Medicine, University of Murcia, Murcia, Spain; ^6^Faculty of Nursing, University of Alberta, Edmonton, AB, Canada; ^7^Cardiovascular and Stroke Strategic Clinical Network, Alberta Health Services, Edmonton, AB, Canada; ^8^Department of Translational Medicine, University of Ferrara, Ferrara, Italy; ^9^University Center for Studies on Gender Medicine, University of Ferrara, Ferrara, Italy; ^10^Division of Clinical Epidemiology, Research Institute of McGill University Health Centre, McGill University, Montreal, QC, Canada

**Keywords:** gender dimension, cardiovascular disease, pulmonary function, chronic kidney disease, gender, sex

## Abstract

Cardio-pulmonary diseases, which were once regarded as a man's illness, have been one of the leading causes of morbidity and mortality for both men and women in many countries in recent years. Both gender and sex influence the functional and structural changes in the human body and therefore play an important role in disease clinical manifestation, treatment choice, and/or response to treatment and prognosis of health outcomes. The gender dimension integrates sex and gender analysis in health sciences and medical research, however, it is still relatively overlooked suggesting the need for empowerment in the medical research community. Latest advances in the field of cardiovascular research have provided supportive evidence that the application of biological variables of sex has led to the understanding that heart disease in females may have different pathophysiology compared to males, particularly in younger adults. It has also resulted in new diagnostic techniques and a better understanding of symptomatology, while gender analysis has informed more appropriate risk stratification and prevention strategies. The existing knowledge in the pulmonary field shows the higher prevalence of pulmonary disorders among females, however, the role of gender as a socio-cultural construct has yet to be explored for the implementation of targeted interventions. The purpose of this review is to introduce the concept of gender dimension and its importance for the cardiopulmonary continuum with a focus on shared pathophysiology and disease presentation in addition to interrelation with chronic kidney disease. The review presents basic knowledge of what gender dimension means, and the application of sex and gender aspects in cardiovascular medicine with a specific focus on early pulmonary development, pulmonary hypertension, and chronic obstructive pulmonary disease (COPD). Early vascular aging and inflammation have been presented as a potential pathophysiological link, with further interactions between the cardiopulmonary continuum and chronic kidney disease. Finally, implications for potential future research have been provided to increase the impact of gender dimension on research excellence that would add value to everybody, foster toward precision medicine and ultimately improve human health.

## Introduction

Non-communicable diseases (NCD) are a growing concern worldwide due to the numerous medical conditions associated with several slow and progressive chronic illnesses involving various organ systems of the body. The term NCD has been expanded to include a broad variety of health issues which include cardiac, pulmonary, renal, gastrointestinal, hepatic, endocrine, and neurologic disorders (1). The human disease process is a complex interaction of different organ systems that has been termed organ-system or organ-body crosstalk (2). This cross-talk between organs contributes to metabolic balance or imbalance such that acute or chronic dysregulations in one organ system may result in dysfunction or failure in another organ (3). With the increasing global burden of cardio-respiratory disorders in terms of mortality and morbidity, a greater effort should thus be made to further expand our knowledge of the interconnections between the vascular system, heart, lung, and kidney. By envisioning a common soil to describe the interconnections between these organ systems, researchers and clinicians worldwide can ensure a comprehensive approach to clinical research endeavors and ultimately enhanced and personalized patient treatment strategies.

This paper, will attempt to address the sex and gender aspects in connection to their relevance in the implementation of contemporary day research. Indeed, as indicated by the European Commission, integrating sex and gender aspects in the research and innovation content presents the concept of gender dimension, which is related to one of the goals of enhancing gender equality balance. The gender dimension takes into account the biological characteristics of both females and males referred to as sex, while the gender domain includes the evolving social and cultural features of women, men, and gender-diverse people.

The current understanding of the cardio-pulmonary continuum in relation to cardiovascular disease, pulmonary hypertension (PH), chronic obstructive pulmonary disease (COPD) in respect to the overlapping factors observed in inflammaging, early vascular aging process as well as cardio-pulmonary-renal interaction will be discussed in relation to research findings according to sex and gender perspectives.

## Importance of sex as a biological variable

Sex pertains to the biological, physiological, and physical differences in genes, hormones, reproductive organs, body fat composition, etc. in males and females. Sex and gender influence disease susceptibility, clinical manifestation, illness severity, treatment choice, therapy responsiveness, health-seeking habits, and prognosis (4). Biological sex and gender factors interact, such that sex for example can influence health by affecting behavior. Estrogen, for example, tends to accelerate the metabolism of nicotine in women, and sex-related changes in nicotine metabolism might alter smoking habits including responsiveness to treatment (5, 6). Differences in metabolism may explain why males respond better to nicotine replacement therapy like patches and gum than women, and men tend to be more susceptible to nicotine's addictive pharmacologic effects (6).

Another female-specific factor that has an impact on health, is the fluctuation of female hormones during the menstrual cycle which can affect mood symptoms (7) and physical performance (8). Pregnancy disorders such as preeclampsia or gestational diabetes, as well as the physical structure of a woman's circulatory system being smaller and more elaborately branched than males, impose a greater risk for future occurrence of cardiovascular disease (CVD) (6, 9). During pregnancy, women are also at an increased risk of bone mass deterioration thus increased bone fragility and osteoporosis (10). The use of hormonal contraceptives in women of reproductive age with migraine may increase the risk for ischemic stroke (11). Women with the polycystic ovarian syndrome (PCOS) have been noted to have emotional disturbance, weight problems, infertility, acne and hirsutism which can thus affect their quality of life (12). Biological sex characteristics and gendered socio-cultural and socioeconomic factors are interlinked and can influence health outcomes in different illness spectra. In ischemic heart illness, a sex difference in clinical presentation is also observed. For example, pain located between the shoulder blades, shortness of breath, nausea, or vomiting, are more frequent in women. Men are more likely to have main vessel obstructive coronary artery disease, while women have coronary microvascular dysfunction leading to the progression of myocardial ischemia (13).

In preclinical and clinical research, an understanding of sex as a biological variable is vital for our comprehension of the basic processes that contribute to disease probability and resilience, especially in circumstances when variations in sex are recognized (14). In experimental studies, including samples from both males and females is important, and sex-disaggregated analysis should be constantly performed. Insufficient inclusion of male or female cell lines and study animals in experiments, disregarding sex-specific data analysis, may contribute to poor reproducibility of biomedical preclinical studies (15). The difference between males and females in respect to illness prevalence, manifestation, and even treatment response may lie in the foundation of genetic influence which begins at conception. The fundamental chromosomal difference of embryos carrying the XX or XY chromosomes results in the variations of the molecular building blocks making up the male and female cells. The idea of asymmetric inheritance is thought to be the source of females' evolutionary advantage in various animal species. For starters, males have only one copy of the X-chromosome, but females have two copies, so any potentially harmful alleles may be obscured by normal alleles on the second X chromosome (16). Another theory based on asymmetric inheritance proposes that there is the optimal selection for compatibility with the female nuclear genome because mitochondria are maternally inherited, and as a result, any hostile male genes would be deleterious to male life expectancy (16). However, biparental inheritance of mitochondrial DNA has been reported in exceptional cases (17), this paper has been highly debated but still worth consideration.

## Gender domain

In studies involving human populations, it is also necessary to consider the socio-cultural complexity consisting of gender relations, gender identity, and gender roles. Both sex and gender impact health and disease, and it is therefore important to appreciate both aspects and their interaction. In daily clinical practice, the physician's principal goal is to provide the best and equal health care to all patients, regardless of ethnicity, background, or socioeconomic level. Every patient is unique, and physicians must recognize and value this uniqueness to achieve the best possible health outcomes. Individuals' health experiences will be determined by their biological sex and gender, which confers cultural and social factors that may operate simultaneously. Being a man or a woman varies between cultures, which is why gender is a distinct factor in health. At times, clinicians, scientists, and service consumers are not always conscious of the consequences of sex or gender perspective in clinical practice or academic research.

**Figure 1:**
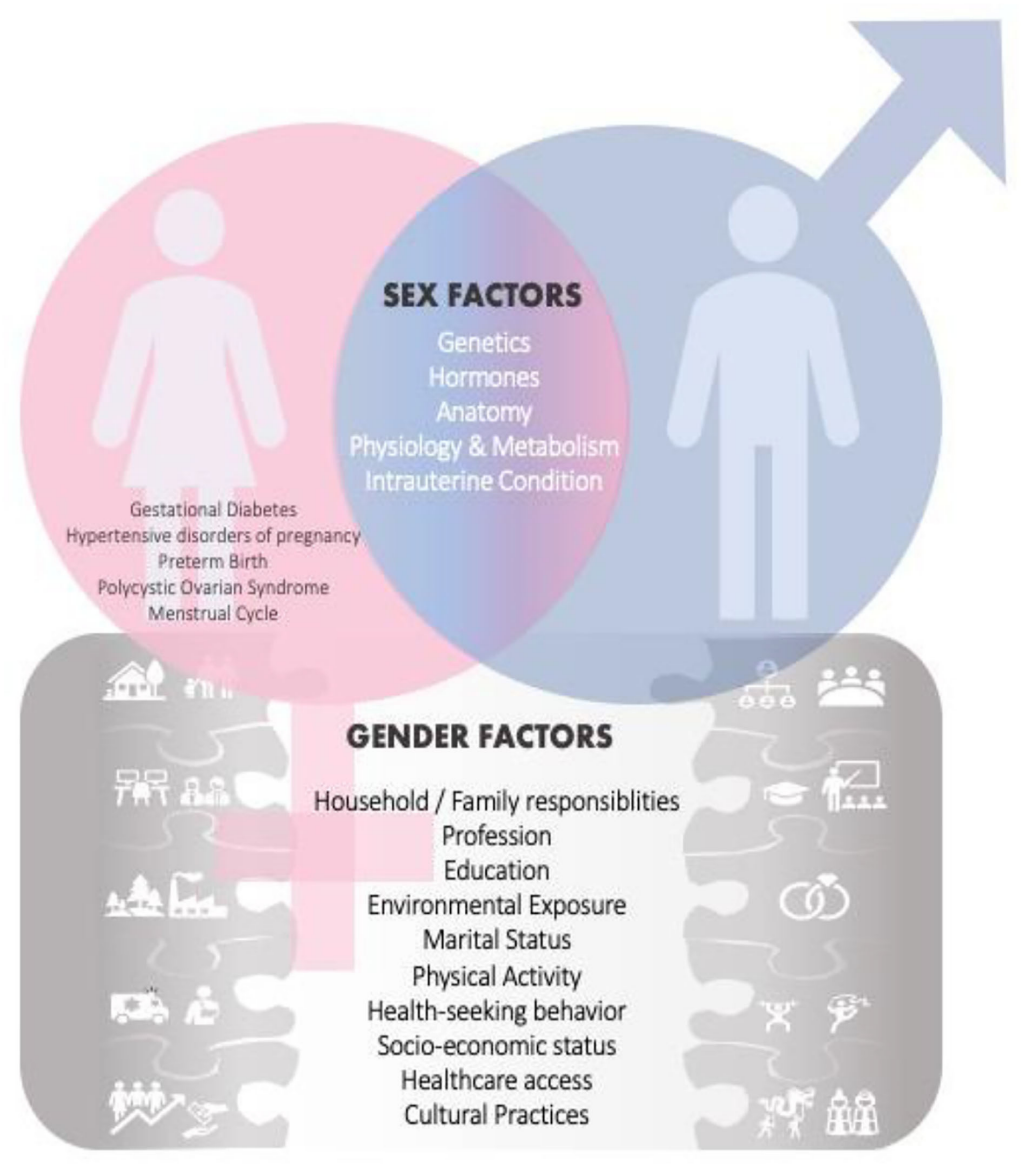
Sex and Gender Factors influence research and health outcomes. In addition to the usual shared sex factors among males and females, there are also sex related factors [i.e., *gestational diabetes, hypertensive disorders of pregnancy, polycystic ovarian syndrome (PCOS), menstrual cycle*] specific only to women. This female-specific factor places a heavier burden on certain diseases in women. Gender factors are also introduced, and those may have an effect on health and disease outcomes.

The gender dimension rationale is integrating sex and gender analysis which enhances the quality of research. It is applying sex-disaggregated analysis in various interdisciplinary areas, and the perspective on gender equilibrium is relevant as it leads to achieving balance and improving the quality of research, innovation, creativity, and excellence (Figure 1). By incorporating sex and gender analysis into experimental design, the scientific discovery would advance in developing better treatment for various diseases and increase social equality and benefits for human health (18). Gender dimension in research denotes also that gender is included in the study design and taken into consideration throughout the research process without necessarily being the primary focus of analysis (19), but nevertheless, be applied wherever practical and relevant.

**Figure 2:**
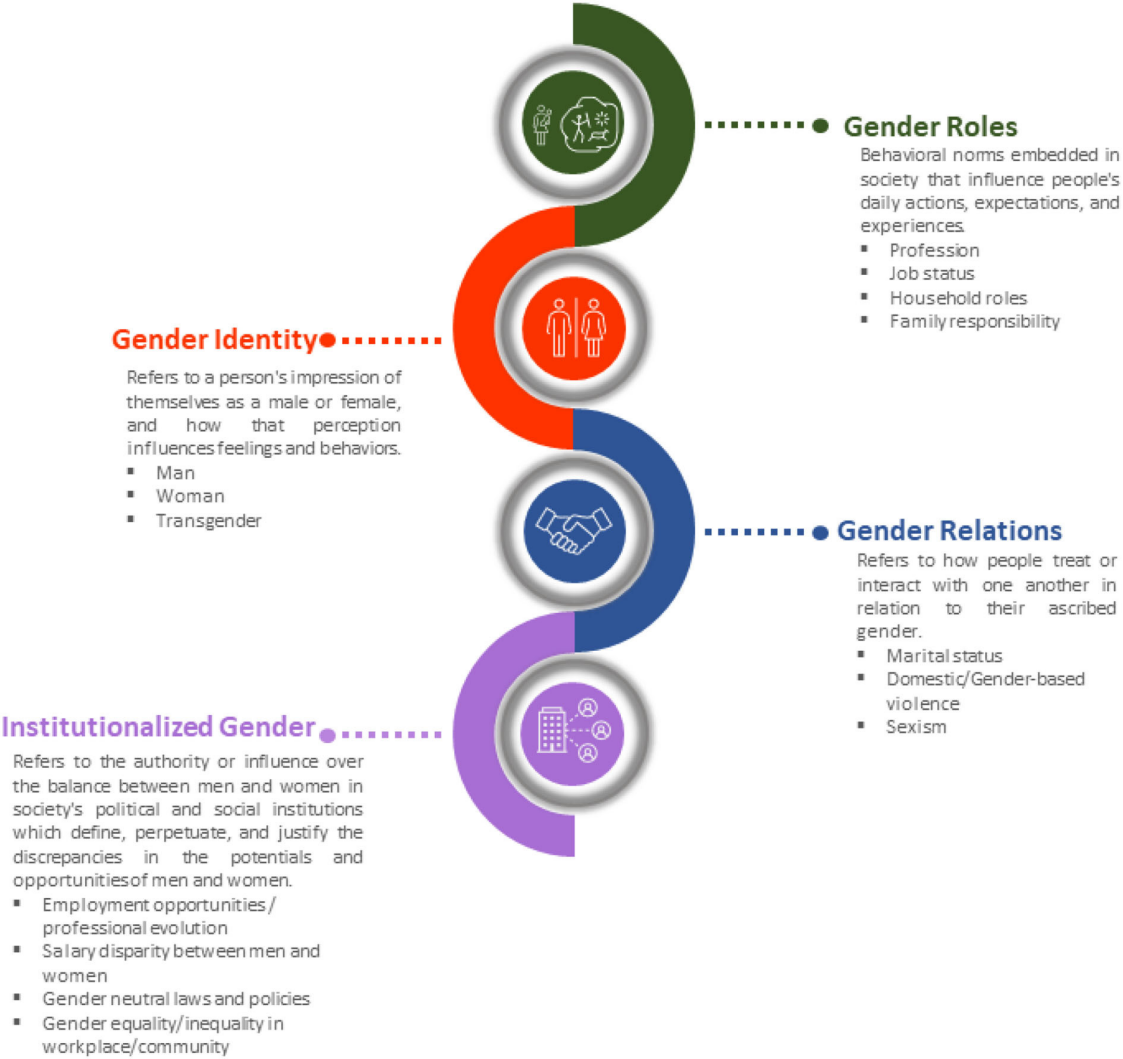
The four gender domain constructs: gender role, gender identity, gender relations, institutionalized gender refers to a person's characteristics or qualities in relation to culturally determined norms.

Males and females are similar in many ways, but they differ significantly in terms of biology and behavior. Gender, a social construct described only in humans refers to attitudes and behaviors associated with what is considered masculine or feminine. In different societies, men, women, boys, and girls are defined by socially and culturally formed norms, values, roles, and expectations (19, 20). There are four domains that comprise the concept of gender (Figure 2), (a) *gender roles* are societal behavioral norms that influence people's everyday activities, expectations, and experiences such as nutrition, smoking, stress, and physical exercise and activity have an impact on health and disease vulnerability; (b) *gender identity* refers to how a person identifies themselves, and how that perception influences feelings and behaviors; (c) *gender relations* denotes how people treat or interrelate with one other according to their assigned gender; (d) *institutionalized gender* refers to the power or control of the balance between men and women in society's political and social institutions, and establishes societal norms defining, perpetuating, and justifying the disparities in women's and men's potentials and opportunities (13, 20).

Gender factors such as stress, environmental exposures, lifestyle choices, or poor nutrition can affect biological parameters and thus health (21). The gender-related characteristics of men and women may have a distinct effect on health, such that bias can exist in the diagnosis and treatment of disease (22, 23). Being regarded as male or female may also elicit distinct reactions from physicians and other medical personnel who may formulate gender-biased diagnoses and management (24). Furthermore, the outcome of health may also be influenced by differences in coping techniques (25) and help-seeking behavior among men and women (26).

Gender medicine advocates for all sexes' needs for better health and healthcare. Thus, doctors and scientists may need to focus on certain areas of medicine or study where data on men and women are limited (21). Sex and gender differences must be addressed, especially in health as a “one-size fits all” approach is less beneficial when it comes to the concept, promotion, and benefit of individualized healthcare measures.

**Figure 3:**
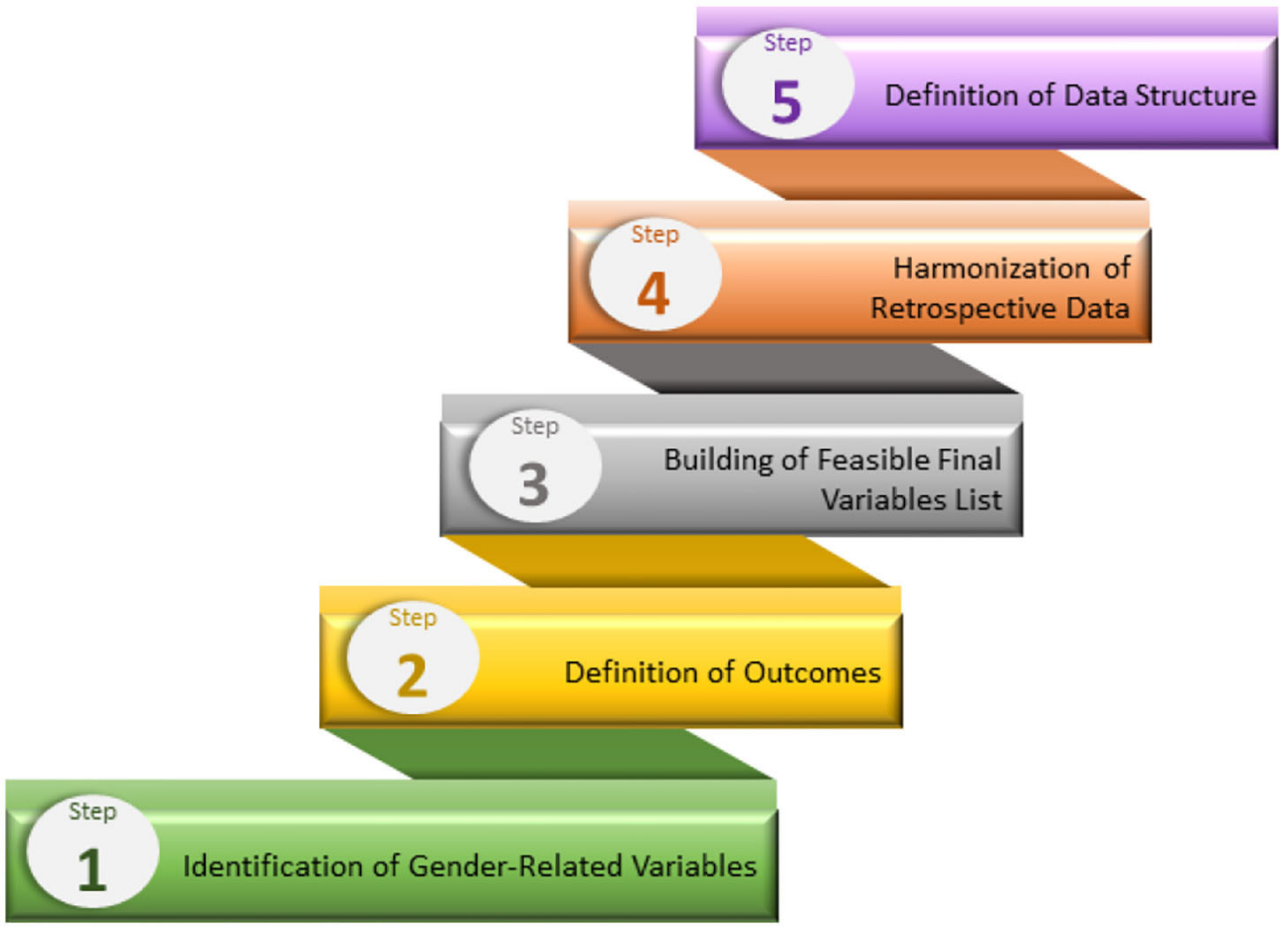
The GOING-FWD multistep approach for identifying and including gender characteristics in research.

As part of the Gender Outcomes International Group: to Further Well-being Development (GOING-FWD) initiative, is a transatlantic collaboration of a multidisciplinary team of researchers from Canada, Sweden, Austria, Spain, and Italy created a standard five-step technique (4) to identify gender-related characteristics and examine their relevance to research outcomes (Figure 3). This multistep approach aims to aid researchers in identifying gender-related factors and how to create a research plan on the core dataset when different variables in datasets are merged. In addition, the GOING-FWD consortium is also an advocate for promoting a patient-partner organization in research, wherein interactions are built on cooperation and mutual respect in all aspects of information dissemination, offering valuable inputs and participation in public forum meetings and other relevant research activities.

## The cardio-pulmonary continuum under sex and gender lenses

### Sex and gender in cardiovascular disease

#### Effects of sex

Cardiovascular disease (CVD), a commonly age-associated ailment oftentimes accompanied by metabolic disturbances, is the leading cause of morbidity and mortality in men and women in industrialized nations (27, 28). The sex differences in the increased risk, pathogenesis, and progression of CVD have been attributed to the sex-related changes in sex hormones, vascular biology, and other cellular changes (29). Males tend to develop CVD at a younger age than women due to the cardioprotective role of estrogen in females (28). This sex difference exists until menopause when the incidence of CVD in females quickly approaches and surpasses that of males correlating with the decline of female sex hormones that are associated with menopause (30). Women after menopause have a 10-fold increase in CVD risk, compared to a 4.6-fold increase in men when comparing the same ages groups (30).

Many symptoms associated with CVD include aberrant vascular control, blood pressure dysregulation, poor tissue perfusion, capillary edema, and inflammatory cell recruitment that could lead to hypertension and atherosclerosis (31). Significant elements in CVD progression are vascular inflammation and endothelial dysfunction (31, 32). Estrogens are important regulators of vasomotor tone. The endothelial cell membrane contains binding sites for estradiol leading to the activation of endothelial nitric oxide synthase (eNOS), which promotes the production of nitric oxide (NO) that causes vascular relaxation and endothelial-dependent vasodilation (28). The decrease of estradiol vasodilatory effects along with unopposed vasoconstriction are responsible for the higher deterioration of endothelial function and related increase in CVD in women postmenopause (28, 33, 34).

While traditional and non-traditional risk factors play a role in the CVD process in both men and women, there are additional female-specific risk factors during the reproductive age that may increase a woman's risk for future CVD, in conditions such as hypertensive disorders of pregnancy, gestational diabetes, premature birth, miscarriage, premature menopause, and PCOS (35). Although premenopausal women have been reported to have lower rates of CVD than age-matched males, inflammation-induced endothelial dysfunction is protected by the female sex because of estrogen resulting in faster resolution of inflammation (31). However, premenopausal women with excess androgens, such as those with PCOS, demonstrate higher levels of inflammatory molecules, develop mild hypertension, oxidative stress, and NF-kB activation, and exhibit features of metabolic syndrome (28, 36).

Understanding the consequences of cardiovascular pathology among males and females is important, nevertheless, transgender persons must also be taken into account, as it has been noted that gender-affirming therapy (GHT) used to align an individual's traits with their gender identity may increase the risk of CVD (37). According to a review by Connelly et al. (37), GHT may increase the risk of CVD due to (a) elevation of BP in both transgender males and females, (b) possible alteration of the thrombotic phenotype, especially in transgender females, or (c) modification in cardiometabolic parameters. Further research is required to comprehend the long-term consequence of GHT and the influence of GHT on CVD risks and occurrences. Regarding the transgender and CVD aspect, it is also essential to recognize that the distribution of the influence of sex and gender on CVD risk in this population may not be easily distinguishable at times owing to the complex and combined impacts of biological, social and cultural aspects.

#### Effects of gender

Gender may interact with biological sex and other social variables such as race, socio-economic status, or behavior to alter cardiovascular health. Indeed the way sex and gender intersect with determinants—such as our income, education, social and physical environments, genetics, and personal health practices—will create varied experiences and health outcomes across populations. (21). A recent study examining the effect of long work hour exposure found that working ≥10 h daily for >10 years was significantly associated with ischemic heart disease (IHD) in men (38). Fadel et al. (38) explained that long work hours may encourage harmful habits associated with an increased risk of CVD, such as smoking, poor nutrition, and inactivity. Another is that chronic psychosocial stress or job shifts may result in activating the autonomic nervous or immune system which may increase the risk for CVD (38). According to Lee et al. (39) sleep deprivation also increases the risk of CVD by reducing productivity and immune function which can lead to social concerns with friends and family. The same group further noted that lack of sleep has been associated with depressive symptoms in older women and death in older men. Sleep quality, duration, and CVD seem to be influenced by sex, and women have been noted to suffer more from insomnia than males due to hormonal changes affecting the circadian rhythm (39).

The quality of preventive care received by a population from its community also has an impact on CVD status. As shown by population-based research in Canada, female immigrants had equivalent or higher overall quality of primary CVD preventive care than males (40). This finding was explained by the possibility that all residents including immigrants are included in the adequate hospital coverage for necessary medical treatments, disease screening, and physician prescriptions. The same group also observed that compared to males, immigrant females from Africa compared to those who came from Caribbean countries used more lipid-lowering and antihyperglycemic drugs. This may in part be due to differences in health-seeking behaviors, customary healthcare practices, and possible inequities in the healthcare system from the country of origin. The social vulnerability has a negative impact on health such that those who are at an increased socioeconomic disadvantage have elevated CVD morbidity and mortality (41).

High education has been linked to lower mortality from all causes, CVD, and coronary heart disease in both sexes (42). Additionally, individuals at high risk for CVD, marital status is independently associated with CVD outcomes, with increased mortality in the unmarried group (43). Age-adjusted mortality from all causes is greater among single people than among married people, both in men and women (42). Furthermore, it has been discovered that individuals who are divorced or separated have a higher risk of myocardial infarction (MI) and cardiovascular mortality than those who are married (43–45). Having a spouse may enhance (modulate) men's lifestyle and treatment compliance thus improving CVD outcome (45). A population-based study in Finland with 302,885 male and female participants showed that men living alone (unmarried, divorced, or widowed) had a higher fatality rate for MI. While in women, cohabitation (living with a non-marital partner) but not marriage was related to a higher fatality (46). This suggests that there is a protective effect on cardiac health associated with living with a marital partner owing to the emotional support from partners. Though cohabitation may offer the same support mechanism as marriage, it may also present greater break-up rates, implying less dedication, stability, and permanence than marriage (46). In addition, a comprehensive understanding of the impacts of marriage or cohabitation on the outcomes of CVD must likewise be considered among same-sex couples, but this may be hampered by a lack of data or studies on same-sex partners and marital quality.

Concerns about CVD must also be addressed in light of the unique medical needs of transgender people (those who were born with a sex that differs from their current gender identification). CVD is one of the health risk factors that transgender people suffer significant variances in prevalence and incidence as well as disparities in outcomes (47). Due to an increase in social pressures, health disparities, and low socioeconomic levels, the transgender group has been reported to have a greater prevalence of behavioral and CVD risk factors compared to the cisgender population (48). The Behavioral Risk Factor Surveillance System (BRFSS) study reported that transgender men have increased odds of having MI when compared to cisgender women and cisgender men, respectively, whereas transgender women had increased odds of having MI when compared to cisgender women but were not significantly different from cisgender men (48). Transgender individuals' increased risk of MI could also be attributed to socioeconomic factors such as depression, stress, unemployment, and poverty, which are often the difficulties faced by the transgender population (49). Numerous transgender individuals are marginalized by stigma, discrimination, violence, and poor health. Accessing proper health care, whether specific to their gender requirements or of a more general character, is frequently difficult for them (50). Challenges to inadequate medical training and clinical guidelines may hamper meeting the unique health care needs of transgender people (47). The existing lack of gender identification in data and minimal research on the transgender community may also limit the application of research results to transgender people.

### Atherosclerosis

Atherosclerosis is the leading cause of CVD and is a progressive inflammatory disease of the vessel wall, which starts a multitude of biochemical and histologic events that are characterized by cholesterol deposition in the arterial wall resulting in atherosclerotic plaque development and subsequent rupture (51). Atherosclerosis plaque development and rupture may cause CVD events including myocardial infarction and stroke (52). Damaged endothelium permits cholesterol-containing low density lipoprotein (LDL) particles to accumulate in the artery wall, triggering an inflammatory reaction that fails to resolve (53). Inside the lipid necrotic core of the atheroma, monocytes develop into macrophages, which subsequently become foam cells. Inflammasomes in macrophages generate interleukin-1 (IL-1), interleukin-18 (IL-18), and other pro-inflammatory cytokines, which are chemotactic toward other inflammatory cells, including B-cells and T-cells, which are significant drivers of atherosclerosis (53). And, because women may have distinct sex-related conditions, such as PCOS, preeclampsia, gestational diabetes, and menopause, they are more likely to develop atherosclerotic cardiovascular disease (9). Carotid artery intima-media thickening (IMT), which is an established marker of atherosclerosis is noted to be increased in women with PCOS, and the PCOS-IMT connection is partly related to associated risk factors and weight and fat distribution (9).

#### Effects of sex

Sex differences have been observed in terms of atherosclerotic plaque development and pathophysiology. Phenotypically, in coronary artery disease, it has been observed that females develop atherosclerotic plaques that are less prone to rupture and of a smaller size when compared to males (54). In addition, in carotid artery disease, an age-specific difference was observed in the likelihood of intraplaque hemorrhage (IPH) being more prevalent in males within younger age groups. However, this sex difference in IPH dissipates in increasing age groups indicative of post-menopause (55). Whilst sex differences in plaque phenotype and size are evident, inflammation within the atherosclerotic plaque may be more relevant in assessing sex-specific mechanisms correlating with CVD mortality (56). Atherosclerotic plaques from males have been found to have significantly increased levels of inflammatory infiltrates, i.e., macrophages, when compared to plaques from age-matched females (57, 58). Interestingly, inflammation-related metabolic activity, assessed by ^18^F-fluorodeoxyglucose (FDG) uptake during positive emission tomography, has been observed at significantly increased levels in the carotid and femoral arteries of males in a cohort of patients undergoing asymptomatic carotid artery screening compared to females, and comparing the metabolically active subjects men were two-fold more likely to have carotid stenosis (59). This indicates that females have less macrophage infiltration and a lower inflammatory contribution within atherosclerotic plaques compared to males, which may be independent of clinical manifestation and CVD risk profile.

#### Effects of gender

Lifestyle habit such as smoking is a substantial risk factor for the development of atherosclerosis, and female smokers are more likely than male smokers to have heart attack (9). Passive smoking or exposure to noxious environmental tobacco smoke may exacerbate risk factors in metabolic syndrome, vascular inflammation, thrombus formation and atherosclerosis (9). According to Amiri et al. (60), there is a dose-dependent connection between CVD and smoking, therefore males who smoke more than women are at higher risk of CVD. However, smoking behaviors and reporting discrepancies between men and women may vary by culture and country. There may be a difference in reporting of smoking habits between men and women in more conservative nations like the Middle East, where smoking is seen as a social shame for women (60).

Low socioeconomic status has been linked to an increased prevalence of atherosclerosis as determined by common carotid IMT and carotid stenosis (61). According to the Swedish study (61), women with less education had a higher risk of carotid stenosis than women with more education, this pattern was weakly observed in men. It was also noted that women in manual occupations also had a higher risk of carotid stenosis than women in non-manual occupations, whereas males had no such risk. Furthermore, there was no association between IMT and occupational status in men, whereas there was in women (61). Rosvall's data suggest that both sexes have atherosclerosis and that there may be sex variations in the mechanisms linking socioeconomic position to atherosclerosis.

Gender factors also influence the acceptability and efficacy of invasive and/or non-invasive cardiovascular diagnostics and medical intervention as strategies to address cardiovascular disease (62). Based on an Australian study, gender differences exist in lifestyle adherence among patients who underwent percutaneous coronary intervention for coronary artery disease (63). Authors noted that women are less likely to be physically active, less likely to attend cardiac rehabilitation, and are less likely to comply with statin therapy compared to men possibly due to a higher occurrence of myalgia among females. Disparities in health behavior, in addition to sociocultural norms imposed by society, contribute to behaviors that may steer the development of premature vascular aging phenotype (please see later chapter) include: risk-taking behaviors such as smoking, poor nutrition, lack of physical activity, stress, noxious environmental factors, socioeconomic disadvantage, and handicap or stigma (62).

## Sex and gender in lung diseases

### Pulmonary hypertension

Definition of pulmonary hypertension (PH) is based on increased mean pulmonary arterial pressure of 20 mmHg or greater. PH is a multifactorial condition and is classified into 5 different subgroups: group 1—pulmonary arterial hypertension (PAH), group 2—PH as a result of left-sided heart disease, group 3—a chronic lung disease caused PH, group 4—chronic thromboembolic PH, group 5—multifactorial/ unclear etiology PH (64). Here we will mainly discuss sex differences in PAH (group 1).

PAH represents pulmonary artery remodeling and dysfunction, and increased pulmonary vascular resistance (64, 65) with predominance among women (65). Increased pulmonary arterial pressure in PAH is polyetiological and includes chronic inflammation, oxidative stress, hormonal disturbances, endothelial dysfunction, and phenotypic switching of pulmonary vascular cells (66). The negative outcome of PAH is right heart failure.

#### Effects of sex

The knowledge that women are more prone to develop PAH is based on data acquired from different national PAH registries (67–73). Meanwhile men with PAH have worse clinical outcomes for 2 and 5 years after PAH diagnosis compared with women as REVEAL (the Registry to Evaluate Early and Long-term PAH Disease Management) reports, and especially at the age of 60 and greater (74). The role of sex hormones in the prevalence and pathogenesis of PAH has been explicitly defined by previous works (65, 75). Indeed, women compared with men who caries *BMPR2* (*bone morphogenetic protein receptor type II*) mutation—inherited as an autosomal dominant trait—have at least two-fold higher risk to get PAH (76, 77). Though the risk for all-cause mortality has been shown to be sex independent (77). Beyond the role of BMPR2 mutation, reduced expression of normal BMPR2 allele could lead to higher PAH predisposition in women (78). Therefore, the interplay between estrogen and BMPR2 signaling (79) advocates for female predominance in PAH manifestation.

It's important to understand that estrogens have a rather contradicting effect on vasoconstriction, vascular smooth cell proliferation and fibrosis—a hallmark of PAH. Estrogens trigger vasodilatation of pulmonary vasculature through NO pathway and have an anti-inflammatory effect on lung tissue (80). Furthermore, the cardioprotective properties of estrogens explain the lower incidence of right heart failure in females (80), however, the possible adverse estrogen influence on pulmonary vasculature in early life must be elucidated by future studies while increasing evidence that environmental chemicals and pollutants with estrogenic features may affect intrauterine development and trigger disease development later in life (81).

Some subgroups of PAH have worse survival, e.g., Porto-pulmonary hypertension (POPH) as compared to idiopathic PAH. POPH is the third most common cause of PAH and is related to liver diseases (82).

#### Effects of gender

Pulmonary Hypertension Association Registry (83) reported that patients suffering from POPH (51% females) as compared to idiopathic PAH (75% females) have lower socioeconomic status because they are more likely to be unemployed, have lower education and lower-income further indirectly suggesting that gender-sensitive environment could have a significant impact on the disease outcome. In support, Wu et al. (84) showed that a lower socioeconomic score determines a worse outcome and higher risk for mortality in idiopathic PAH, where the study population was up to 80% consisting of females. Hopefully, further investigations would address more in detail the importance of sex and gender aspects in those analyses.

### Chronic obstructive pulmonary disease

#### Effects of sex

Chronic obstructive pulmonary disease (COPD) is recognized as a disease with a chronic inflammatory state that is characterized by progressive infiltration of innate and adaptive inflammatory leukocytes in the lungs. In addition, C-reactive protein (CRP), necrosis factor-alpha (TNFα), interleukin-6 (IL-6), pulmonary and activation-regulated chemokine (PARC), vascular endothelial growth factor (VEGF), and tumor matrix metalloproteinase 9 (MMP-9), have been linked to the development of COPD (85). COPD patients have higher vascular leakage than healthy patients, VEGF regulates the formation of new arteries, and men have higher levels of VEGF and IL-6 than women in COPD patients (85). COPD is reported to be of two primary types: chronic asthmatic bronchitis which has more female preponderance, and emphysema which is more frequently noted in males (86). Women are less likely than males to die after being admitted to the hospital for COPD, and if they would need artificial ventilation, their survival rate is lower than those of males (87). Due to cyclical hormonal swings, women may perceive variation of symptoms, even noting diminished respiratory function differently than men thus may experience and report shortness of breath more than males, which is a symptom of impaired ventilatory performance and a COPD and emphysema clinical feature (87).

#### Effects of gender

COPD was once thought to be a condition primarily affecting elderly males but sex difference in COPD exists with regards to susceptibility, smoking status and exposure, presence of co-morbidities, and prevailing underdiagnosis in women (88). In recent years, the incidence of COPD in women has risen, thus narrowing the gap between men and women (89). Possible reasons include women's increased tobacco use and exposure, longer life expectancy among females, and occupational and non-occupational exposure (85, 90). Socio-economic and socio-cultural factors may contribute to the sex discrepancy in COPD leading to gender differences in respiratory symptom awareness, presentation, and diagnosis. For example, Townsend et al. (91) reported that almost 80% of those diagnosed with COPD were non-smoker females which may relate to second-hand exposure in women from smoker male partners.

In addition, the greater risk for hospitalization of women with COPD may likewise be due to women's increased tendencies to seek medical help compared to men (91). Increasing patient awareness of COPD also improves self-management efficiency. One research has reported (92) that females lacked knowledge on how to execute a good action plan and what to do in an emergency. Insufficient patient information may lead therefore to higher exacerbations and emergency visits.

## Sex and gender-aspects during preconception, early life factors and lung development

Both the intrinsic and extrinsic environmental factors during pre-conception, intrauterine, and postnatal stages play a role in respiratory health's vulnerability to future potential adult respiratory disorders (93, 94). Suboptimal environmental factors during a key time of lung development may affect a person's respiratory health due to altered lung structure, and function, as well as maladaptive responses to hazardous agents which may result in impaired lung function in adulthood (95).

For example, cigarette smoke exposure, preterm birth and nutrition, birth weight, and early exposure to viruses and allergens are all possible environmental variables that can affect the likelihood of respiratory illness later in life (95). Prenatal and perinatal exposure to air pollutants can result in various unfavorable outcomes like preterm birth and low birth weight (LBW) (96). Developmental defects in lung function early in life are associated with future lung diseases and impaired respiratory function that can extend to adolescence and adulthood (96). Males and females may experience varied impacts to their respiratory health from poor intrauterine conditions and exposure to hazardous substances according to an extensive review by Harding's group (93). Maternal smoking impairs the lung function of offspring due to the influence of nicotine that can rapidly cross the placenta and affect the pulmonary fibroblasts' role in alveolarization (93). Other smoking-related impacts include permanent reduction of energy metabolism in the lungs, as well as increased primitive alveoli size and volume, which reduces gas exchange (93). Airway resistance is higher in female fetuses exposed to maternal smoking, this smoke exposure “masculinizes” the female fetal airways, causing lower airflow rates comparable to males (97). Cigarette smoke exposure during pregnancy and as well as during breastfeeding can increase airway hyperresponsiveness and neutrophil penetration into the lungs, as well as alter the immunological response in the offspring, according to studies in mice (98).

Male and female differences in lung development, susceptibility, pathogenesis, morbidity, and mortality to certain respiratory disorders may be influenced by genetic, hormonal, and environmental variables before and during the neonatal period (99). Androgen and estrogen receptors are expressed in the human lung (99, 100), and male lung maturation typically lags throughout the fetal period due to the delayed production of surfactants secondary to the inhibitory effect of Mullerian-inhibiting hormone from male fetal testes-derived Sertoli cells (101). Compared to females, surfactant production is stimulated by estrogen that favors lung maturation (85, 99, 102). Also, a more abundant surfactant in the lungs of female neonates encourages patency of tiny airways and airspaces contributing to better flow rate and lesser airway resistance (91, 99). Airway development in girls is proportionate to lung parenchymal growth compared to boys wherein airway maturity trails behind parenchymal development causing a disparity between airway and lung size which increases airway resistance (97, 101).

Boys and girls have the same number of alveoli per unit area and alveolar volume, but boys have larger lungs than girls (91, 99). Males have lower forced expiratory flows than females due to more smooth muscle and broader airways, and airway resistance grows quicker in males than in females as they mature (97). The difference in pubertal patterns of lung development has been observed between sexes, as noted in asthma there is male predominance until puberty after which there is a switch resulting in higher incidence in females during adulthood (91, 101).

Preterm-born neonates when compared to those born at term are associated with increased respiratory problems, higher frequency of airflow blockage, gas entrapment, and impaired lung gas exchange (85, 103). Preterm birth has been linked with increased respiratory symptoms notably respiratory disease syndrome (RDS) and bronchopulmonary dysplasia (BPD) which explains the significant share of the morbidity and mortality among prematurely born infants (104). RDS is characterized by surfactant deficiency (105), while BPD is exhibited by a halted alveolarization process and abnormal development in pulmonary vasculature in preterm infants (85).

Sex disparities in morbidity and mortality between prematurely born male and female neonates exist, with males having a significant disadvantage compared to females born at comparable gestational age (106). Male babies are more likely than female neonates to have a higher risk of developing RDS and dying from it (99). Females have a lower incidence of RDS than males regardless of race or ethnicity (107). Before the introduction of glucocorticoids and surfactant therapy, RDS was a major cause of neonatal mortality among male neonates (85). The type of prenatal glucocorticoid (dexamethasone vs. betamethasone) used to prevent RDS was reported to exhibit a sex-specific result (108). Despite the use of mechanical ventilators, prenatal corticosteroids, and surfactants to minimize morbidity and death in very low birth weight newborns, there are still variations in outcomes that favor females compared to males (109). Prematurely born infants who develop BPD can develop long-term consequences such as recurrent or severe respiratory problems (110), pulmonary arterial hypertension (85), and poor neurodevelopmental outcomes particularly in low-birth-weight male neonates (111).

As mentioned earlier, one factor for the increased risk for RDS associated with males can be accounted from the hormonal influence during fetal lung development. Aside from early surfactant production, another advantage of preterm females is the stronger Na^+^ transport driven alveolar fluid clearance (AFC) due to higher mRNA expression of epithelial Na^+^ transport channel (ENaC) and Na, K-ATPase, which may be facilitated by improved sensitivity to female sex hormones through higher estrogen receptor-β (ER-β) and progesterone receptor (PR) expression (112).

Sex hormones regulate lung function maturation, with androgens acting as inhibitors while estrogens as stimulators. The placenta produces estrogen, whereas the fetal testes secrete testosterone (113). Estrogens' positive regulatory function in lung development is particularly prominent during the latter stages of lung development namely the saccular to the alveolar stage (102). Increased estrogen concentrations in female fetuses boost estrogen receptor activation, which upregulates genes that influence morphological, functional lung development, and surfactant homeostasis (102). Animal studies using male and female mice have shown that there is direct transcriptional regulation of granulocyte-macrophage colony-stimulating factor (GM-CSF) and platelet-derived growth factor-alpha (PDGF-α) by estrogens *via* ER-β receptor (114). Patrone's findings suggest a biological explanation for the observed sex disparities in adult lung alveolar shape and identify estrogen receptors in lung development and homeostasis, although further studies are needed to understand lung development and estrogen receptors and their role after birth. Estrogen receptors ER-α and ER-β are necessary for alveoli maintenance according to studies in female mice (115). The expression of ER-β protein in the human bronchial epithelial cells is double that of ER-α (116), and in knockout ER-α and ER-β mice, both types of ER are essential for the alveolar unit development (117). ER-α facilitates appropriate lung differentiation, which results in normal alveoli per surface area numbers, while ER-β, regulates the growth of the extracellular matrix resulting in the lung's flexible tissue recoil pressure (117). Further studies are important to understand the complexity of the hormone environment interaction with hormone receptors after birth and whether it has an impact on the development of lung disease.

## Inflammageing, a common pathway for understanding sex and gender based aspects of CVD and lung diseases

### Effects of sex

The human body typically has its core physiological defense mechanisms against foreign pathogens or tissue injuries. However, the complicated reaction triggered by a shift from normal physiological body function to non-resolving pathological processes such as the continuous and protracted presence of inflammation can be harmful to health (118). The term “inflamm-aging” or” inflammageing” is a term coined by Claudio Franceschi, it is defined as an age-related disorder characterized by increased levels of inflammatory markers in the circulation such as IL-1β, IL-6, TNF-α (119) and hsCRP (high-sensitivity CRP) (120). These pro-inflammatory mediators in the blood increase the risk for chronic illnesses, disability, frailty, and death (53, 121). Activation of the NLRP3 (NOD-, LRR- and pyrin domain-containing protein 3) inflammasome, oxidative stress induced by malfunctioning mitochondria, dysregulated immune cells, and chronic infections are all possible pathways of inflammageing (53). Inflammageing has also been linked to other conditions such as CKD (122, 123), atherosclerosis, diabetes, cancer, depression, dementia (118) as well as lung changes and intestinal dysbiosis that occur with advancing age (119). Inflammation has been reported to be a most probable causative risk factor as well as a pathogenic process for CVD such that inflammation influences CVD, multimorbidity, and frailty by suppressing growth factors, increasing catabolism, and disrupting homeostatic signaling (53).

One factor that may play an important role in inflammation is the sex-associated hormonal change resulting in the observed disparities in inflammation between males and females. In SARS-CoV-2 infection, females are protected against viral infections due to X-chromosomes and sex hormones which regulate adaptive and innate immunity (124). COVID-19 is associated with a greater risk of serious illness and death in men as women are more protected due to estrogen which increases innate and humoral immune responses thus lessening the severity and fatality of COVID-19 (124). A previous study (120) found that women had a stronger association than males in IL-6 and hsCRP hs-CRP levels and that the 51–60 age range appeared to be a pivotal point for this increase. Women's estrogen-mediated regulation of inflammatory biomarkers changes with age, and there are enhanced ER-β expression and pro-inflammatory effects of estrogen that occurs with aging (32). Novella et al. (32) reported that endogenous estrogen depletion contributes to postmenopausal women's age-related escalation in CVD risk. Thus, the cardiovascular protective benefits of estrogen medication propose that the benefits of estrogen in preventing CVD may emerge only when therapy commences before the detrimental effects of aging on the vasculature are evident (32). After age 65, disparities between sexes also become more pronounced, with men exhibiting more innate and pro-inflammatory activity and less adaptive immunity (125). Bupp et al. (126) noted that the aging process is associated with changes in the immune system's function, that inflammaging may cause dysregulation of innate immune cells further adding to inflammatory processes. Men and women have quite distinct immune systems, and that aging may confer a sex-specific effect on the immune system's cellular composition and function which in turn affect age- and sex-associated changes in the frequency and process of autoimmunity, cancer, vaccination efficiency and cancer immunotherapy (126). Immunosenescence is marked by weakened immune capabilities, and inflammaging results in a shortage of anti-inflammatory mediators (127). Thus, immunosenescence and inflammaging when combined play a critical role in the decay of the immune system to combat diseases such as SARS-CoV-2 infection thus resulting in severe COVID-19 in elderly patients (127).

### Effects of gender

Aside from molecular or cellular processes, gendered variables such as human behavior can also potentially confer a protective or damaging effect on health. For example, physical exercise or active lifestyle behavior has been reported to influence inflammation by inducing an anti-inflammatory effect by reducing toll-like receptor (TLR) expression on monocytes and changing the phenotype of macrophages in adipose tissue (128). In the young population, gender differences are observed in exercise behaviors, motivations for exercise, and quality of life (129). Inflammaging increases the susceptibility to poor prognosis due to the dysfunctional systemic immune response, which is manifested as a cytokine storm in SARS-CoV-2 infection. According to Padilha de Lima et al. (130), in the COVID-19 situation, strategies that promote an overall healthy lifestyle should be emphasized for both sexes. These strategies include healthier dietary modification, increased physical activity, as decreased physical activity has a detrimental effect on muscle quality and poor nutritional habits which can accelerate immunosenescence and inflammation (130), and in the longer term accelerate arterial stiffening (131).

Women reported having a higher quality of life than men, and they tend to exercise more for weight loss and toning, while men exercised more for fun (129). However, for older people, the pattern and level of physical activity may be limited by the risk or occurrence of injury particularly falling. In a study by Stahl and Albert (132) older females were more prone to being frequent fallers in comparison to older males but did not seem to have an effect on their participation in leisure activities or domestic work, while older males who experienced frequent falls resulted in decreased recreational/ leisure activities and household labor. The authors explained that women participated in relatively little leisure activity which may account for why falling had no effect on their activity pattern. Furthermore, the difference between males and females may be attributed to their gender role expectations for household chores such as females performing more caregiving activities (132). Physical activity is a significant and controllable risk factor for severe COVID-19 outcomes, according to Sallis et al. (133). When compared to individuals who were persistently inactive, those who engaged in some physical activity had a lower chance of hospitalization and mortality, indicating that any level of physical activity in both women and men might help minimize the risk of dying from COVID-19 (133).

## Early vascular aging: Sex and gender

Early vascular aging (EVA) is a progressing concept that undiagnosed arterial stiffness that may contribute to an earlier onset of CVD and subsequently an increase in mortality (134). The combination of various damaging stressors to the artery wall, as well as the time spent exposed, might accelerate the normal vascular aging process. EVA is characterized by an increased peripheral vascular resistance due to a decreased elasticity of the arteries (52). Another pathophysiological link to CVD is endothelial dysfunction which could further impair vasodilation resulting in arteriosclerosis phenotype. Endothelial cells in the microvasculature play a role in immunological responses, inflammation, thrombosis, remodeling, and arteriolar vessel tone modulation (135). Vascular endothelial cell damage associated with atherosclerosis releases antigens that initiate and prolong inflammatory responses, and senescent cells are commonly seen in considerable amounts in atherosclerotic plaques (53).

EVA related endothelial senescence and dysfunction may cause pulmonary artery remodeling (136). Evidence from animal studies shows that senescent cells in lung vasculature fuel the progression of pulmonary artery hypertension (137), however the precise outcome of those investigations could not be related to a specific sex, as the sex-disaggregated analysis was not performed. Additionally to these findings, premature microvascular damage due to endothelial dysfunction could contribute to the interrelationship between pulmonary function/diseases and higher risk for CVD (138, 139). According to an investigation of patients with COPD (71% male study group), patients with COPD have a decreased vasodilatory response when compared to controls, which correlates to increased cardiovascular morbidity (139).

The appearance of EVA has been identified in different lung conditions. Increased cardio-ankle vascular index (CAVI), a proxy of arterial stiffness, was associated with pulmonary age in hypertensive patients (140). The other study observed borderline differences in magnetic resonance acquired systemic pulse wave velocity in COPD as compared to healthy controls (141). The early signs of crosstalk between systemic vasculature and lung function are already prevalent in childhood as seen in an investigation of 8-year old children—a study population composed of 50% females—observed a higher carotid augmentation index as well as lower lung volumes (142).

As for the sex differences, at least two studies (143, 144) have shown that males in comparison to females have stiffer arteries, assessed by carotid-femoral pulse wave velocity (cfPWV), which could help to predict the loss of lung function in male subjects. The Swedish CArdioPulmonary bioImage Study (SCAPIS) (145) observed the reduction in PWV by 1-SD increase in Forced Expiratory Volume 1 sec (FEV1) and diffusing capacity for carbon monoxide only in males but not in females from healthy populations.

Thus, resolving the sex and gender issues in early vascular aging and pulmonary health may provide novel targets for modifying vascular remodeling and developing arterial destiffening as well as targets for slowing lung fibrosis.

In a female-specific condition such as PCOS and in the presence of lower lung function (146) development of EVA could be linked with increased CVD later in life (147). This supports the suggestion that metabolic syndrome is encompassed by a combination of factors responsible for EVA such as obesity, hyperglycemia, dyslipidemia, and hypertension (148, 149) further presenting a higher female predominance (150). Aside from that, other mechanisms, e.g., increased plasma visfatin concentrations, have been shown to be associated with CVD in PCOS as compared to healthy obese females (151).

Lungs and vascular tree share similar structural properties, though, the interaction between systemic and pulmonary vascular dysfunction are not fully understood (141). The theory that inflammaging drives these two processes in COPD seems to be plausible, and telomere attrition may be a missing detail in this puzzle. Indeed, telomere shortening has been linked to cellular senescence and CVD (152, 153) as well as pulmonary diseases (154). Telomeres are positioned at the ends of the chromosomes which are essential for chromosomal maintenance. When cell divides telomeres replicate incompletely and subsequently cause DNA damage. Telomere lengths have been noted to be sex-specific, with males being born with shorter telomeres than females (155). Males are programmed to have faster telomere shortening as compared to women (156). Given that women live longer than males, telomeres have been proposed as the causative explanation for the disparity (155). However, telomere attrition in both sexes in combination with other factors, such as oxidative stress or inflammation, triggers cellular senescence (152). The combination of the above-mentioned cellular processes, including variables such as lifestyle choices, physical exercise, and food, might alter vascular repair mechanisms, and likewise affect telomere length (157). Reduced cell damage and endothelial dysfunction, and longer telomeres have been linked to a healthy lifestyle which includes eating a diet rich in fruits and vegetables, particularly the Mediterranean diet, exercising regularly, maintaining a low body mass, and avoiding smoking which are important factors in lowering the risk of cardiovascular disease (158). In a Spanish population study, it was observed that adherence to the Mediterranean diet or consumption of a healthy diet is influenced by occupational socioeconomic strata, particularly younger women who are in danger of social exclusion (159). The authors however could not conclude whether the greater cost of particular Mediterranean diet staples such as fish and fruit is the major reason for the disparity in adherence. Further studies are warranted indeed to assess those gender-sensitive aspects in the aging process.

## Cardio-pulmonary-renal interactions

Organ cross-talk is a concept used to describe and explain various health conditions. It helps to understand complex pathogenetic pathways behind multi-organ failure. The interplay between heart and kidney has been recently clarified and classified into 5 different subtypes mainly stressing bidirectional interactions (160). Interestingly, the fifth type defines simultaneous acute dysfunction of both heart and kidney in systemic conditions such as sepsis. As for pulmonary-renal syndrome, it often represents a respiratory failure—diffuse alveolar hemorrhage or acute respiratory distress syndrome (ARDS)—in combination with renal failure—glomerulonephritis or acute kidney injury without present hematuria (161). The most common causes that account for 68.5–95% of all cases are anti-glomerular basement membrane (anti-GBM) disease antineutrophil cytoplasm antibody (ANCA)—associated vasculitis (162, 163). Both diseases have well-defined sex differences in prevalence and outcomes (164) with clear predominance among men.

Unfortunately, the Cardio-Pulmonary-Renal interaction is not well investigated considering its burden of mortality. This is also valid for the absence of indications of sex and gender perspectives. In the light of extended pandemics with SARS-CoV-2 infection, the importance of ARDS in the Cardio-Pulmonary-Renal continuum is worth commenting. Indeed, ARDS is a pro-inflammatory condition (165) associated with critical illness due to determining alterations in the alveolar-capillary barrier with following need for mechanical ventilation. Therefore, here some aspects of sex differences could be of interest. For example; a large observational study (166) showed that shorter women were more likely to receive lower tidal ventilation and that the female sex was associated with higher mortality in severe ARDS. The iatrogenic trauma to the lungs affects pulmonary microcirculation that furtherly leads to increased right ventricular afterload and impaired cardiac output. Finally, it may result in reduced renal flow and acute ischemic kidney failure (167).

The sex differences in immune response (168) might play an important role in different ARDS presentations alongside insufficient ventilation regimes and admission to the intensive care unit. The pathogenesis of ARDS involves mechanisms related to innate and adaptive immunity (169) that consequently will affect target organs such as the heart and kidney. Alveolar epithelial cells, macrophages, and dendritic cells are the first barriers responding to diverse triggers and resulting in cellular response and damage (169), most probably in a sex-specific way, due supportive evidence of sex-specific phenotypes of these cells as seen in animal and human studies (170). For example, females may have a thicker airway wall most likely due to greater protein processing and its phosphorylation in the endoplasmic reticulum in addition to active protein folding (170).

Phelps et al. (171) investigated the mechanisms behind changes in alveolar macrophage activity in the presence or absence of Surfactant Protein-A (SP-A). The SP-A modulates host-defense lung function, including its potential to act as an opsonin assisting in the clearance of different pathogens by alveolar macrophages. Proteomic analysis demonstrated an increase in numerous alveolar macrophage protein spots, suggesting that SP-A may have unique features in females vs. males, since several of the discovered proteins affected estrogen activity (171). Under infectious conditions, SP-A enhances alveolar macrophages immune response which may explain why males could have higher incidence of bacterial pneumonia (172).

At this stage it is also worth noticing the importance of interaction between PH and CKD, and if sex *per se* could have an effect on both conditions' development. Indeed, PH and CKD could share underlying causes such as autoimmune connective tissue diseases seen predominantly in females and immunodeficiency virus-associated disease in males (173). However, neither a retrospective study of Chinese subjects (174) nor a report from the USA (175) had suggested the predominance of one sex for increased risk in PH in CKD. An additional study reported that in CKD stages 3 to 5 there is a 1.4-fold higher risk for PH (50% male subjects) (176), further indicating increased mortality from PH in males with CKD. Yet, further studies with identification of sex and gender aspects in the disease development and organ interaction are of utmost importance for the combination of PH with CKD.

There are a few literature reports addressing the relationship between COPD and CKD, as it is perceived that CVD is the most important co-morbid that exists in COPD patients. However, further attention must also be placed on the link between between CKD and COPD. The prevalence of CKD and COPD is observed to increase with age and are likewise correlated with atherosclerotic disease, and CKD prevalence in COPD patients ranges from 20–30% (177). CKD and COPD patients share common risk factors such as hypertensive disorders and diabetes (178). According to the German COPD and Systemic Consequences—Comorbidities Network (COSYCONET), poor outcomes may be mediated by CKD's impact on clinical symptoms, functional status, and capacity for exercise. CKD and COPD's share common systemic symptoms like malnutrition, muscle wasting, anemia, osteoporosis, and cardiovascular disease, which all have a negative impact on patients (179). COPD which has been defined as the pulmonary component of systemic endothelial disease wherein the inflammageing process concurrently impact several organs, resulting in a condition of multicomorbidity, without a clear evidence which illness occurred first (180). The inflammatory condition and response observed in both CKD and COPD may exacerbate the symptoms in a bidirectional process. Studies on the repercussions of COPD on CKD or *vice versa* are still limited. One report showed that females (178) with COPD had 93% higher risk of developing CKD as compared to 43% increased risk in males. Another study has also supported the higher incidence of CKD in females with COPD (181). However, the incidence of end-stage renal disease (182) in subjects with COPD is similar between the sexes. Further studies are warranted to understand both sex or gender-related influence on both conditions and their interaction.

An increased number of dendritic cells and the inflammatory response has been identified in female mice model of bronchial asthma (183) which may explain possible underlying causes for the higher prevalence of asthma in women. Hereby inflammation *per se* (184) as well as pro-apoptotic pathways, may contribute to progression toward Cardio-Pulmonary-Renal syndrome in a sex-specific manner, however gendered-specific variables and their impact have yet to be explored in future studies. Bronchial asthma also has an effect on other chronic disorders, particularly those affecting the cardiovascular and glucose metabolic systems. In a Chinese population study, individuals with bronchial asthma (60% female participants) may be at a higher risk of developing CKD, that there is a 9.6% prevalence of CKD in the group with chronic asthma than the group without persistent asthma (185).

According to an extensive review by Hussain-Syed's group (167), understanding the intricate biological transmission and response mechanism between the heart, lung and kidney relationship can serve to better investigate the pathophysiology, epidemiology, diagnosis and therapeutic management of various diseases. A dysfunction in each organ may grow gradually until a cumulative effect ensues or individuals with disease in one organ may die from complications of the other organ before the complete collapse of the first organ (167). Each defective organ is capable of initiating and perpetuating mutual harm through various sex-specific cell signaling feedback or physiologic mechanisms while numerous bouts of acute or chronic decompensatory processes may result in corresponding end-organ-damage. Gender specific variables on the other hand may impact the development of a particular organ disease concurrently, and health outcomes are strongly influenced by human behavior which varies according to societal roles, identity, and other cultural interactions.

## Future directions—targets for intervensions

Biomedical scientists, researchers, and clinicians must have a good grasp of the concepts of sex and gender in research and be able to address all of the important biological and social elements to obtain a scientifically sound result that is valid for human health. There is an important need to further raise awareness in conducting sex and gender-balanced approach in scientific study, and likewise in the use of the “sex” and “gender” terminologies appropriately.

Discussed in some papers are guides (4, 20, 186, 187) for researchers to aid in integrating sex and gender in various phases of research. Incorporating sex and gender into research and innovation contributes to the advancement of scientific knowledge. Consideration of the needs of men, women, boys, girls, and other gender varied factors can improve the quality of disseminated information as well as increase the societal applicability of research findings (188). For example, the approach utilized by Pelletier and the group (189), is to use gender-related scoring in research to produce a femininity or masculinity score that is used to evaluate the recurrence of an acute coronary syndrome.

A sex-informed framework especially in the study of humans considers sex variations in anatomy and physiology, as well as insights on critical times of prenatal, childhood, pubertal development, reproductive window and aging (187). When designing research, it is also critical to evaluate if the investigator has gender preconceptions that may influence the selection of study participants (190). The current situation in the European Union, National Institutes of Health in the United States as well as the Canadian Institutes of Health Research seems to be optimistic as a growing number of professional organizations, funding agencies, philanthropic groups, editors of peer-reviewed journals, academic institutions, ethics boards, health care systems and industry are refining policies and making concerted efforts to integrate sex and gender into biomedical research (191).

Gender study in the laboratory using cell and animal models is somewhat more complicated due to the normally controlled environmental conditions and other practical limitations, and gender is intrinsically a cultural construct in human society that cannot be simply equated with social dynamics among non-human subjects (186). Therefore, it is equally important to disclose and justify the use of single-sex experimental models in research as it may prompt a discourse about the ramifications of certain developments or study outcomes.

The close interconnected nature and many overlapping components addressing the issues of sex and gender pose a challenge for researchers to address them separately. At times, a variety of gender-related variables may inhibit or exacerbate inherent biological inequalities in health (4). The interaction between sex and gender may influence the probability of acquiring certain illnesses, symptoms and prognosis, and response to treatment (4). Currently, several articles (4, 192, 193) have been published to provide a practical guide for researchers on how to integrate sex and gender into their studies.

Resolving the sex and gender issues in methodologies or research designs may provide novel insights that are crucial for the generation of new ideas or hypotheses, as well as increasing the relevance and practical application of results and their reproducibility. It cannot be overemphasized that research must move toward sex and gender-balanced research. Equal representation of both sexes including the social constructs that accompany any study population of interest would further enhance the advancement of targeted precision medicine in improving human health and corresponding therapeutic management. Efficient analysis of disparities between males and females would pave the way to further identify gaps in information and comprehend how and why the differences occur in order to create effective strategies to remedy them.

Finally in respect to human diagnosis and therapy, it is known that males and females have a propensity for increased burden in certain disease areas. However, in the majority of cases, males and females tend to be subjected to the same medical management as most clinical trials have been skewed toward males, and underpowered in female participants. Therefore, we must recognize the importance and need for a system where both sexes get balanced representations to get equally safe treatments. In the era of modern medicine, clinicians must have a clear and comprehensive awareness of the necessity of providing customized medicine to both sexes (194). As noted by Tannenbaum and Day (194) comparable drug trial findings across age and sex groups from improved research designs, data collecting, selection of biomarkers and analytic methods will undeniably improve risk-benefit profiling for the identification of new age-or sex-specific pharmacological targets.

## Author contributions

KK contributed to the conceptualization of this review paper together with LH, AL-C, and LW for review layout. LH, AL-C, LW, and KK drafted the manuscript. AK-W, M-TH, CN, VR, LP, PS, and KK critically reviewed the manuscript. All authors read and approved the final manuscript.

## Funding

The review was supported by the Njurfonden (Swedish Renal Foundation) (PS and KK), Hjärt lungfonden (PS), CIMED (PS), ALF (PS), and Swedish Research Council (Vetenskapsrådet 2018-00932 GOING-FWD) (KK).

## Conflict of interest

Author PS serves on the Scientific Advisory Boards of Baxter, Astra Zeneca, and REATA. The remaining authors declare that the research was conducted in the absence of any commercial or financial relationships that could be construed as a potential conflict of interest.

## Publisher's note

All claims expressed in this article are solely those of the authors and do not necessarily represent those of their affiliated organizations, or those of the publisher, the editors and the reviewers. Any product that may be evaluated in this article, or claim that may be made by its manufacturer, is not guaranteed or endorsed by the publisher.

## Abbreviations

AFC: Alveolar fluid clearance
ANCA: Antineutrophil cytoplasm antibody
anti-GBM: Anti-glomerular basement membrane
ARDS: Acute respiratory distress syndrome
BMPR2: Bone morphogenetic protein receptor type II
BPD: Bronchopulmonary dysplasia
CAVI: Cardio-ankle vascular index
cfPWV: Carotid-femoral pulse wave velocity
CKD: Chronic Kidney Disease
COPD: Chronic Obstructive Pulmonary Disease
COSYCONET: COPD and Systemic Consequences &#8211
Comorbidities Network
CRP: C-reactive protein
CVD: Cardiovascular Disease
ER-β: Estrogen receptor-beta
ENaC: Epithelial Na^+^ transport channel
EVA: Early vascular aging
FDG: Fluorodeoxyglucose
FEV1: Forced Expiratory Volume 1 s
GM-CSF: Granulocyte-macrophage colony-stimulating factor
hsCRP: high-sensitivity CRP
IHD: Ischemic Heart Disease
IL: Interleukin
IPH: Intraplaque hemorrhage
IMT: Intima-media thickening
LBW: Low birth weight
LDL: Low density lipoprotein
MMP-9: Matrix metalloproteinase 9
MI: Myocardial infarction
NCD: Non-communicable disease
NO: Nitric oxide
PCOS: Polycystic ovarian syndrome
PAH: Pulmonary arterial hypertension
PARC: pulmonary and activation-regulated chemokine
PDGF-α: Platelet-derived growth factor-alpha
PH: Pulmonary Hypertension
POPH: Porto-pulmonary hypertension
PR: Progesterone receptor
RDS: Respiratory disease syndrome
REVEAL: Registry to Evaluate Early and Long-term PAH Disease Management
SP-A: Surfactant Protein-A
SCAPIS: Swedish CArdioPulmonary bioImage Study
TLR: Toll-like receptor
TNFα: Tumor ne necrosis factor-alpha
VEGF: Vascular endothelial growth factor.
